# ADDRESSING THE HIGH RISK OF OPIOID OVERDOSE AMONG OLDER ADULTS: A RETROSPECTIVE CHART REVIEW

**DOI:** 10.1093/geroni/igae098.4375

**Published:** 2024-12-31

**Authors:** Bethea Kleykamp, Erin Lynch, Hannah Smith

**Affiliations:** University of Maryland School of Medicine, Baltimore, Maryland, United States; University of Maryland School of Medicine, Baltimore, Maryland, United States; University of Maryland School of Medicine, Baltimore, Maryland, United States

## Abstract

**Background and Objectives:**

In Maryland, fatalities due to opioid overdoses are highest among adults aged 55 and over a. This retrospective chart review evaluated 49 adults in outpatient opioid treatment in Baltimore as a function of age. Analyses: Intake characteristics and treatment outcomes were compared between 24 older (ages 55-70) and 25 younger (ages 23-35) adults using ANOVA and χ2 statistics (alpha=0.05).

**Results:**

The cohort was 54% male, and most patients were prescribed methadone (84%). Older adults were more likely to be Black/African American (68% vs. 24%) and had longer histories of opioid (30 vs. 9 years) and tobacco use (45 vs. 14 years) (ps < 0.05). At intake, equal proportions of older and younger adults tested positive for cocaine (49%), fentanyl (61%), and benzodiazepines (27%); cannabis use was less common for older adults (17% vs. 56%; χ2 =8.15). While engaged in treatment, older adults were less likely to test positive for cocaine (26% vs. 77%) and cannabis (16% vs. 60%) (ps < 0.05), with no differences in fentanyl (66%) or benzodiazepine use (29%). Older adults remained in treatment longer (186 vs. 105 days, F=6.7) and had higher retention rates (21% vs. 0%) (χ2 =5.8).

**Conclusion:**

Older adults showed less polysubstance use and longer retention in care. However, continued fentanyl and benzodiazepine use could put older adults at greater risk of overdose due to age-related vulnerabilities and prolonged substance use. These differences highlight the unique clinical characteristics of older adults at risk of overdose, which can inform age-friendly addiction treatment approaches.

